# Short course of antimicrobial treatment for uncomplicated enterococcal bacteremia

**DOI:** 10.1007/s10096-025-05348-6

**Published:** 2025-11-07

**Authors:** Virgile Zimmermann, Nicolas Fourré, Laurence Senn, Benoit Guery, Matthaios Papadimitriou-Olivgeris

**Affiliations:** 1 Infectious Diseases Service, Lausanne University Hospital and University of Lausanne, Lausanne, Switzerland; 2 Infection Prevention and Control Unit, Lausanne University Hospital and University of Lausanne, Lausanne, Switzerland; 3 Infectious Diseases Service, Hospital of Valais and Institut Central des Hôpitaux, Sion, 1951 Switzerland

**Keywords:** Enterococci, Uncomplicated bacteremia, Sepsis, Shorter is better, Duration of antimicrobial treatment, *Enterococcus faecalis*

## Abstract

**Purpose:**

Duration of treatment for uncomplicated enterococcal bacteremia is unknown. This study aims to evaluate the clinical outcomes of patients treated with short courses (4–10 days) compared to those receiving longer regimens (11–18 days).

**Methods:**

This retrospective study was conducted at the Lausanne University Hospital, Switzerland (January 2015-June 2024) and included adult patients with uncomplicated enterococcal bacteremia. Primary outcome was a composite of mortality, recurrence of bacteremia by the same enterococcal species and development of bone and joint infection within 120 days.

**Results:**

During the study period, 331 episodes of uncomplicated enterococcal bacteremia were included. The median duration of antimicrobial treatment was 12 days (interquartile range: 8–15); 138 (42%) and 193 (58%) episodes received a short (4–10 days) and long (11–18 days) duration of antimicrobial treatment, respectively. The primary endpoint was observed in 77 (23%) episodes; 120-day mortality was 21% (69 episodes), recurrence of bacteremia was 4% (12 episodes), and bone and joint infection was 0.6% (2 episodes). No difference in primary endpoint was observed between episodes receiving short and long courses of antimicrobial treatment (23% *versus* 23%; *P =* 1.000). The Cox multivariable regression model found that malignancy (aHR 2.00, 95% CI 1.24–3.22), immunosuppression (1.78, 1.09–2.90), cirrhosis (2.53, 1.42–4.51), and sepsis or septic shock (2.48, 1.52–4.03) were associated with primary endpoint; a short course of antimicrobial treatment was not associated with primary endpoint (1.03, 0.65–1.62).

**Conclusion:**

Among uncomplicated enterococcal bacteremia giving a short duration of antimicrobial treatment is reasonable.

**Supplementary Information:**

The online version contains supplementary material available at 10.1007/s10096-025-05348-6.

## Introduction

Bacteremia remains a critical public health challenge, associated with substantial morbidity and mortality [1, 2]. Historically, most bacteremias were treated with antimicrobial regimens lasting at least 14 days [3, 4]. Over the past two decades, multiple randomized controlled trials, primarily addressing Gram-negative bacteremias, have compared short-duration treatments with traditional longer courses, consistently demonstrating similar efficacy and safety between the two approaches [5, 6].

In contrast, studies examining treatment duration for bacteremia caused by Gram-positive cocci are limited, and predominantly focus on *Staphylococcus aureus*bacteremia [7, 8]. Current guidelines and narrative reviews recommend a treatment duration of 7–14 days for uncomplicated enterococcal bacteremia [9, 10]. Retrospective studies have reported a median treatment duration of 10–14 days for these cases [11–14]. Only two studies have assessed the impact of treatment duration on outcomes in uncomplicated enterococcal bacteremia, both concluding that short courses were safe and not associated with treatment failure [11, 12]. However, these studies were limited to catheter-related infections and were subject to allocation bias, as shorter antibiotic durations were typically prescribed for healthier patients. The randomized BALANCE trial, which included 3,608 cases of bacteremia, with 250 attributed to enterococci, demonstrated that a 7-day antibiotic regimen for uncomplicated bacteremia caused by pathogens other than *S. aureus*was non-inferior to a 14-day regimen; [6] however, no pathogen-specific analysis was conducted for the enterococcal cases.

To address the lack of evidence regarding shorter durations of antimicrobial treatment for all uncomplicated enterococcal bacteremias, this study aims to evaluate the clinical outcomes of patients treated with short courses compared to those receiving longer regimens.

## Materials and methods

This retrospective study was conducted at Lausanne University Hospital, Switzerland from January 2015 to June 2024 combining two cohorts: the retrospective bacteremia cohort (January 2015 to December 2021), and the prospective cohort of patients with suspected IE (January 2022 to June 2024).

Inclusion criteria were adults (≥ 18 years old) with at least one blood culture for *Enterococcus* spp. and absence of patient’s decline of the use of their data. Exclusion criteria were incomplete medical records (transfer to other hospital without follow-up in our institution), absence of initiation of appropriate antimicrobial treatment within 48 h from first positive blood culture, death or instauration of palliative care within 14 days from the first positive blood culture, infection classified as complicated (those requiring a *de facto* duration of treatment longer than 10 days) and delay of the decision on total duration of antimicrobial treatment after day nine. Criteria for complicated disease were: infective endocarditis, bone and joint infection, deep vein septic thrombophlebitis, vascular graft infections, prostatitis, persistent bacteremia for at least 48 h, neutropenia, clinical instability at day five of bacteremia onset, polymicrobial bacteremia involving *S. aureus* or *Candida* spp., and lack of warranted source control interventions within 48 h.

Blood cultures were processed using the BacT/ALERT System (bioMerieux, Marcy l’Etoile, France) and species identification was performed using matrix-assisted laser desorption-ionization time of flight mass spectrometry (MALDI-TOF MS; Bruker Daltonics, Bremen, Germany). Susceptibility results were assessed in accordance with the EUCAST criteria [15].

Data were extracted from electronic medical records, encompassing demographics (age, sex), comorbidities, Charlson Comorbidity Index, antimicrobial treatment (intravenous, oral, duration), adverse events (allergic reactions, interstitial nephritis, and *Clostridioides difficile* infection), source control, presence of sepsis or septic shock, and site of infection were retrieved from patients’ electronic health records by internal medicine and infectious diseases (ID) consultants. All data were reviewed by an ID consultant.

In our institution, ID consultants were informed for positive blood cultures after species identification. For enterococcal bacteremia, ID consultation was not mandatory. Decisions on cardiac imaging studies were guided by the ID consultant, or left to the treating physician’s discretion if no ID consultation was provided [2].

The primary endpoint included mortality, recurrence of bacteremia by the same enterococcal species and the development of bone and joint infection within 120 days from the first positive blood culture. Short and long course of antimicrobial treatment was defined as 4–10 days, and 11–18 days, respectively. The date of collection of the first positive blood culture was defined as the onset of bacteremia. A new episode was included if more than 120 days had elapsed since the initial bacteremia. Bacteremia was classified as community, healthcare-associated, or nosocomial based on Friedman et al. [16]. Sepsis and septic shock were defined in accordance with the criteria proposed by the Sepsis-3 International Consensus [17]. The infection focus was determined by the ID consultants responsible for the case or, if no ID consultation was provided, by the treating physician based on clinical, radiological, microbiological, and surgical findings. Clinical stability by day five was characterized by hemodynamic stability, absence of fever, and no supplemental oxygen requirement. Documentation of the decision on the total treatment duration was recorded in ID or treating physician notes. Warranted source control measures included removal of venous catheter in patients with catheter-related bacteremia or bacteremia of unknown origin with the presence of a venous catheter, imaging-guided or surgical drainage of infected collections, and correction of urinary-tract obstruction.

SPSS version 26.0 (SPSS, Chicago, IL, USA) were used for data analyses. Fisher exact test or *chi*-square were used for categorical variables and Mann–Whitney *U* test for continuous ones. Univariate logistic regression models were assessed with 120-day primary endpoint as dependent variable. Clinically relevant non collinear covariates, assessed through variance inflation factor, were used in multivariable analysis. After checking Cox assumptions, a multivariable Cox proportional hazards regression models were performed with 120-day primary endpoint as the time-to-event. Adjusted hazard ratios (aHRs) and 95% confidence intervals (CIs) were calculated to evaluate the strength of any association. All statistic tests were 2-tailed and *P* < 0.05 was considered statistically significant. We finally performed Kaplan-Meier curves of the primary endpoint free probability according to duration of antimicrobial treatment.

## Results

**Fig. 1:**
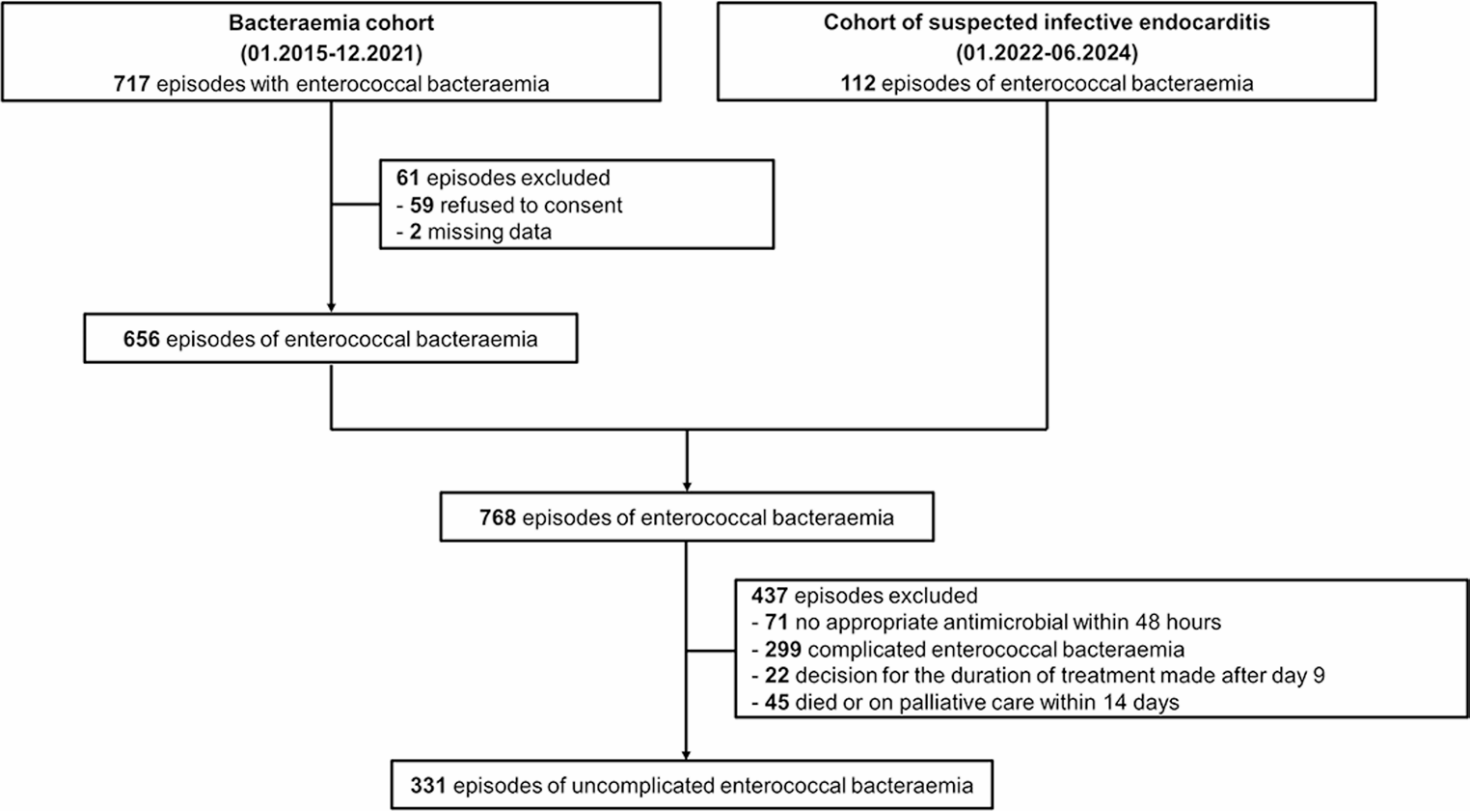
Flowchart of patients’ selection

Of the 768 enterococcal bacteremia episodes, 331 episodes involving 303 patients were included (Fig. 1). From the bacteremia cohort (656 episodes), 283 (43%) had uncomplicated bacteremia. *E. faecalis* was the most frequently isolated species, accounting for 187 (56%) episodes. Resistance to ampicillin and vancomycin was observed in 113 (34%) and 15 (5%) isolates, respectively; all vancomycin-resistant isolates carried the *vanA* gene. Abdominal (139; 42%) and urinary-tract (81; 24%) were the most common foci of infection. Sepsis or septic shock occurred in 108 (45%) episodes. Follow-up blood cultures were performed in 260 (79%) episodes.

Cardiac imaging to rule out infective endocarditis was performed in 158 (48%) episodes, with the first imaging exam conducted at a median of 3 days (interquartile range [IQR]: 1–4 days) and the full diagnostic evaluation completed within a median of 4 days (IQR: 2–6 days). Transthoracic, transesophageal echocardiography, and ^18^F-Fluorodeoxyglucose Positron Emission Tomography/Computed Tomography were performed in 152 (46%), 29 (9%), and 15 (5%) episodes, respectively, with 158 (48%) episodes undergoing at least one type of cardiac imaging.

The median duration of antimicrobial treatment was 12 days (IQR: 8–15), with 138 (42%) episodes receiving short course (4–10 days) of antimicrobial treatment and 193 (58%) episodes receiving long course (11–18 days). Table 1 shows the comparison of the episodes that received short and long courses of antimicrobial treatment. The median duration of intravenous antimicrobial treatment was 9 days (IQR: 7–14). Step down oral antimicrobial treatment was implemented in 66 (20%) episodes. Supplementary Table 1 shows details on the antimicrobial agents used for intravenous and oral therapy. Nine (3%) episodes presented *C. difficile* infection, 5 (2%) interstitial nephritis, and one (0.3%) allergic reaction.

**Table 1:** Comparison of episodes with short (4–10 days) and long duration of antimicrobial treatment (11–18 days)

	Short duration (*n* = 138)	Long duration (*n* = 193)	*P*
Demographics			
Male sex	94 (68)	139 (72)	0.465
Age (years)	70 (57–81)	72 (61–80)	0.369
Age > 60 years	94 (68)	146 (76)	0.136
Co-morbidities			
Diabetes mellitus	36 (26)	63 (36)	0.224
Obesity (body mass index ≥ 30 kg/m^2^)	29 (21)	39 (20)	0.891
Chronic kidney disease (moderate or severe)	29 (21)	58 (30)	0.076
Malignancy (solid organ or hematologic)	45 (32)	63 (32)	1.000
Immunosuppression^a^	32 (23)	57 (30)	0.211
Chronic obstructive pulmonary disease	14 (10)	19 (10)	1.000
Cirrhosis	13 (9)	20 (10)	0.854
Congestive heart failure	11 (8)	11 (6)	0.503
Charlson Comorbidity Index	5 (3–7)	6 (4–8)	0.221
Charlson Comorbidity Index > 4	79 (57)	120 (62)	0.426
Indwelling material			
Prosthetic valve	10 (7)	11 (6)	0.649
Cardiac implantable electronic device	9 (7)	20 (10)	0.244
Vascular graft	6 (4)	12 (6)	0.624
Arthroplasty	25 (18)	26 (14)	0.281
Setting of bacteremia onset			
Community	32 (23)	36 (19)	
Healthcare-associated	19 (14)	37 (19)	
Nosocomial	87 (63)	120 (62)	1.000
Microbiological data			
Two or more positive blood culture sets	55 (40)	106 (55)	0.008
Pathogens			
*E. faecalis*	64 (46)	123 (64)	0.002
* E. faecium*	63 (46)	74 (38)	0.213
Non-*faecalis*, non-*faecium* enterococci	16 (12)	3 (2)	< 0.001
Polymicrobial bloodstream infection^b^	44 (32)	49 (25)	0.216
Ampicillin-resistant	49 (36)	64 (33)	0.724
Vancomycin-resistant	12 (9)	3 (2)	0.003
Follow-up blood cultures	108 (79)	152 (79)	1.000
Infection data			
Fever	119 (86)	166 (86)	1.000
Sepsis or septic shock	68 (49)	80 (42)	0.179
Septic shock alone	21 (15)	23 (12)	0.414
Intensive Care Unit admission	31 (23)	20 (10)	0.003
Focus of infection			
Unknown focus	15 (11)	13 (7)	0.230
Catheter-related	32 (23)	29 (15)	0.063
Abdominal infection	73 (53)	66 (34)	0.001
Urinary-tract infection	12 (9)	69 (36)	< 0.001
Other focus	8 (6)	15 (8)	0.520
Management			
Infectious diseases consultation	104 (75)	163 (85)	0.048
Infectious diseases consultation within 48 h	91 (66)	148 (77)	0.035
Source control			
Not warranted	60 (44)	88 (46)	
Warranted and performed within 48 h	78 (57)	105 (54)	0.737
Antimicrobial treatment			
Initiation before blood culture results	106 (77)	148 (77)	1.000
Step down oral treatment	15 (11)	51 (26)	< 0.001
Duration of IV antimicrobial treatment	8 (6–9)	14 (13–16)	< 0.001
Total duration of antimicrobial treatment	8 (5–9)	13 (10–15)	< 0.001
Adverse events			
Allergic reaction	1 (0.7)	0 (0)	0.417
Interstitial nephritis	1 (0.7)	4 (2.1)	0.406
* Clostridioides difficile* infection within 120 days	2 (1)	7 (4)	0.314
Duration of hospitalization	15 (9–27)	15 (8–28)	0.648
Mortality at 30 days	12 (9)	17 (9)	1.000
Mortality at 60 days	21 (15)	25 (13)	0.629
Mortality at 90 days	28 (20)	34 (18)	0.569
Primary endpoint at 120 days	32 (23)	45 (23)	1.000
Mortality	29 (21)	40 (21)	1.000
Recurrence of bacteremia with the same organism	4 (3)	8 (4)	0.767
New bone and joint infection	1 (0.7)	1 (0.5)	1.000

Data are depicted as number (%) or median (interquartile range)

^a^ongoing immunosuppressive treatment at bacteremia onset, intravenous chemotherapy in the 30 days prior to bacteremia onset, AIDS, and asplenia

^b^11 with multiple enterococcal species, 22 with other Gram-positive bacteria, and 74 with Gram-negative bacteria

The primary endpoint, comprising 120-day mortality, recurrence of bacteremia caused by the same enterococcal species, or development of bone and joint infections, was observed in 77 (23%) episodes; 69 episodes (21%) resulted in mortality, 12 (4%) had recurrence of bacteremia, and 2 (0.6%) developed bone and joint infection (Supplementary Table 2). There was no difference in primary endpoint between episodes treated with short and long courses of antimicrobial treatment in the whole cohort (23% *versus* 23%; *P =* 1.000), among the 187 episodes with *E. faecalis* bacteremia (23% *versus* 21%; *P =* 0.714), or the 155 episodes with bacteremia by other enterococcal species (24% *versus* 25%; *P =* 1.000) (Supplementary Table 3). Table 2 shows the comparison of episodes meeting and not meeting the primary endpoint. The Cox multivariable regression model (Table 3) identified malignancy (solid organ or hematologic) (*P* = 0.004; aHR 2.00, 95% CI 1.24–3.22), immunosuppression (*P* = 0.021; aHR 1.78, 95% CI 1.09–2.90), cirrhosis (*P* = 0.002; aHR 2.53, 95% CI 1.42–4.51), and sepsis or septic shock (*P* < 0.001; aHR 2.48, 95% CI 1.52–4.03) as factors associated with the primary endpoint. The duration of antimicrobial therapy was not associated with the primary endpoint (*P* 0.913; aHR 1.03, 95% CI 0.65–1.62).

**Table 2:** Comparison of episodes that did and did not Meet the primary endpoint (mortality, recurrence of enterococcal bacteremia and bone and joint infection within 120 days)

	No primary endpoint (*n* = 254)	Primary endpoint (*n* = 77)	*P*
Demographics			
Male sex	173 (68)	60 (78)	0.117
Age (years)	71 (59–80)	74 (58–81)	0.566
Age > 60 years	185 (73)	55 (71)	0.884
Co-morbidities			
Diabetes mellitus	72 (28)	27 (35)	0.260
Obesity (body mass index ≥ 30 kg/m^2^)	49 (19)	19 (25)	0.335
Chronic kidney disease (moderate or severe)	65 (26)	22 (29)	0.658
Malignancy (solid organ or hematologic)	69 (27)	39 (51)	< 0.001
Immunosuppression^a^	61 (24)	28 (36)	0.040
Chronic obstructive pulmonary disease	22 (9)	11 (14)	0.191
Cirrhosis	18 (7)	15 (20)	0.004
Congestive heart failure	15 (6)	7 (9)	0.307
Charlson Comorbidity Index	5 (3–7)	7 (5–9)	< 0.001
Charlson Comorbidity Index > 4	140 (55)	59 (77)	0.001
Indwelling material			
Prosthetic valve	19 (8)	2 (3)	0.181
Cardiac implantable electronic device	25 (10)	4 (5)	0.255
Vascular graft	17 (7)	1 (1)	0.085
Arthroplasty	38 (15)	13 (17)	0.719
Setting of bacteremia onset			
Community	57 (22)	11 (14)	
Healthcare-associated	44 (17)	12 (16)	
Nosocomial	153 (60)	54 (70)	0.139
Microbiological data			
Two or more positive blood culture sets	122 (48)	39 (51)	0.698
Pathogens			
*E. faecalis*	146 (58)	41 (53)	0.515
* E. faecium*	103 (41)	34 (44)	0.599
Non-*faecalis*, non-*faecium* enterococci	14 (6)	5 (7)	0.781
Polymicrobial bloodstream infection^b^	73 (29)	20 (26)	0.667
Ampicillin-resistant	81 (32)	32 (42)	0.132
Vancomycin-resistant	12 (5)	3 (4)	1.000
Follow-up blood cultures	198 (78)	62 (81)	0.752
Infection data			
Fever	223 (88)	62 (81)	0.131
Sepsis or septic shock	98 (39)	50 (65)	< 0.001
Septic shock alone	30 (12)	14 (18)	0.179
Intensive Care Unit admission	37 (15)	14 (18)	0.472
Focus of infection			
Unknown focus	19 (8)	9 (12)	0.248
Catheter-related	48 (19)	13 (17)	0.740
Abdominal infection	111 (44)	28 (36)	0.292
Urinary-tract infection	62 (24)	19 (25)	1.000
Other focus	15 (6)	8 (10)	0.201
Management			
Infectious diseases consultation	207 (82)	60 (80)	0.511
Infectious diseases consultation within 48 h	187 (74)	52 (68)	0.311
Source control			
Not warranted	113 (45)	35 (46)	
Warranted and performed within 48 h	141 (56)	42 (55)	0.897
Antimicrobial treatment			
Initiation before blood culture results	194 (77)	60 (78)	0.878
Duration of antimicrobial treatment			
Long duration	148 (58)	45 (58)	1.000
Short duration	106 (42)	32 (42)	

Data are depicted as number (%) or median (interquartile range)

^a^ongoing immunosuppressive treatment at bacteremia onset, intravenous chemotherapy in the 30 days prior to bacteremia onset, AIDS, and asplenia

^b^11 with multiple enterococcal species, 22 with other Gram-positive bacteria, and 74 with Gram-negative bacteria

**Table 3:** Cox proportional hazard multivariable regression of 120-day composite endpoint (mortality, recurrence of bacteremia, new bone and joint infection) among patients with uncomplicated enterococcal bacteremia

	*P*	aHR (95% CI)
Malignancy (solid organ or hematologic)	0.004	2.00 (1.24–3.22)
Immunosuppression^a^	0.021	1.78 (1.09–2.90)
Cirrhosis	0.002	2.53 (1.42–4.51)
Charlson Comorbidity Index > 4	0.069	1.67 (0.96–2.90)
Sepsis or septic shock	< 0.001	2.48 (1.52–4.03)
Short duration of treatment	0.913	1.03 (0.65–1.62)

CI: confidence interval; aHR: adjusted hazard ratio

^a^ongoing immunosuppressive treatment at bacteremia onset, intravenous chemotherapy in the 30 days prior to bacteremia onset, AIDS, and asplenia

**Fig. 2:**
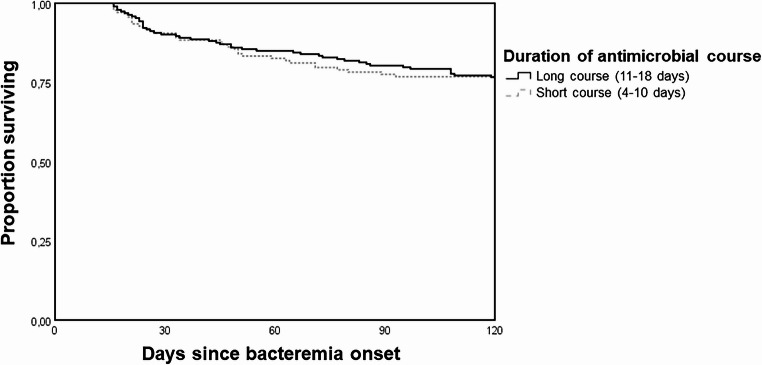
Kaplan–Meier curves for primary endpoint free probability for episodes with uncomplicated enterococcal bacteremia based on the duration of antimicrobial treatment (log-rank test: *P* = 0.916)

Kaplan–Meier analysis (Fig. 2) showed no significant difference in primary endpoint-free survival between short and long course antimicrobial treatment groups for episodes of uncomplicated enterococcal bacteremia (log-rank test: *P* = 0.916).

## Discussion

Among cases of uncomplicated enterococcal bacteremia no significant difference in clinical failure was observed between patients treated with short or long courses of antimicrobial treatment.

The median duration of antimicrobial treatment was 12 days, aligning with previous reports (median duration of 10–15 days) [11–14, 18]. Current guidelines typically recommend 7–14 days of treatment, [9, 10] but practices vary. A survey among European professionals, 85% of which were ID consultants or clinical microbiologists, reported a median treatment duration of 10 days (interquartile range: 10–14) [19]. Practices vary between countries or specialties [3, 4]. ID consultants from Germany treated for 10 days on average compared to 14 days in France [3]. Meanwhile, in Australia and New Zealand, ID consultants tended to prescribe longer courses than intensive care physicians.[4] Similarly, in the present study, longer courses were more common when patients had ID consultation (85%*versus* 75%; *P* = 0.048).

Only two prior studies specifically evaluated the impact of treatment duration on outcomes in enterococcal catheter-related bacteremia, reporting median durations of 11 and 12 days [11, 12]. These studies defined short courses differently: 7 days in the study by Bahrs et al. [11] and 9 days in the study by Marschall et al. [12] with short-course treatment used in 26% and 36% of cases, respectively. In contrast, the present study defined short courses as ≤ 10 days, with 42% of patients receiving this regimen. Both earlier studies concluded that short courses were safe and not linked to treatment failure, including mortality or bacteremia recurrence, though their retrospective design introduced allocation bias favoring healthier patients having received shorter courses [11, 12]. To address this limitation, the current study included only cases where clinical stability was achieved within five days of the initial positive blood culture and treatment duration decisions were finalized by day seven. The randomized BALANCE trial, which investigated uncomplicated bacteremias from pathogens excluding *S. aureus*, demonstrated noninferiority of 7-day versus 14-day antibiotic regimens, but was dominated by Gram-negative pathogens, with only 250 enterococcal cases and no pathogen-specific analysis [6].

In the present study, 120-day mortality (21%) was lower than that reported in previous studies (90-day mortality: 30–45%) [11, 12, 20, 21]. In addition to including patients who achieved clinical stability within five days, we also ensured the inclusion of those who received appropriate antimicrobial treatment within 48 h and who underwent necessary source control procedures within the same timeframe. Timely source control interventions have been previously demonstrated to significantly improve outcomes across various types of infections [22–26]. Moreover, the lower incidence of VRE (5%) in this study, compared to previous studies (34–100%), also played a role in the observed improved outcomes [12, 20, 21]. Consistent with previous findings, malignancy, immunosuppression, and cirrhosis were linked to poorer long-term outcomes, highlighting the influence of underlying comorbidities on the prognosis of patients who survived the initial period.[27, 28]However, an initial presentation with sepsis or septic shock, despite achieving clinical stability within five days, was associated with poorer long-term outcomes, aligning with findings from previous studies [29, 30].

Bacteremia recurrence within 120 days occurred in 4% of cases, with no significant difference between short- and long-course antimicrobial treatment. This rate was lower than previously reported recurrence rates (7–11% over 90 days) [12, 20, 21]. The same factors contributing to lower mortality likely influenced the reduced recurrence rates. Patients with uncomplicated *E. faecalis* bacteremia more frequently received longer durations of antimicrobial treatment, compared to non-*E. faecalis* species. This difference likely reflects the higher perceived risk of infective endocarditis with *E. faecalis*, despite negative diagnostic workup for endocarditis [28]. Notably, only two cases of bone and joint infections were observed within 120 days, and no cases of infective endocarditis were identified. These findings challenge the rationale for prolonged antimicrobial treatment in uncomplicated bacteremia due to non-staphylococcal Gram-positive cocci, which is often driven by concerns about recurrence or metastatic infections, as seen with*S. aureus*bacteremia [31].

This study has several limitations. It was a retrospective, single-center analysis with a modest sample size, though to the best of our knowledge, it represents the largest study to date on duration of treatment of uncomplicated enterococcal bacteremia and the only one not exclusively focused on catheter-related cases. Moreover, the setting in a tertiary care center with high rates of ID consultation limits generalizability. Furthermore, decisions on treatment duration may have been influenced by undocumented factors. However, patient selection was based on stringent clinical criteria, including early clinical stability, prompt source control, and finalized treatment plans by day nine. To mitigate immortal time bias inherent to retrospective studies, cases of early death or transition to palliative care within 14 days were excluded. Despite these precautions, randomized controlled trials remain essential. Currently, a multicenter, open-label, randomized trial (INTENSE) is ongoing, aiming to demonstrate that a 7-day regimen is non-inferior to 14 days for treating uncomplicated enterococcal bacteremia [32].

In conclusion, this study suggests that shorter antimicrobial courses for uncomplicated enterococcal bacteremia is a reasonable option, with no differences in long-term mortality, recurrence, metastatic infections, or treatment-related adverse events between short- and long-course regimens. These findings contribute to the growing body of evidence suggesting that antimicrobial treatment can be safely shortened in a carefully selected subset of patients with uncomplicated disease. Patients presenting with sepsis remained at higher risk of poor long-term outcomes despite initial good response. Standardized definitions of uncomplicated enterococcal bacteremia and randomized trials are urgently needed to refine treatment duration recommendations.

## Supplementary Information

Below is the link to the electronic supplementary material.

Supplementary Material 1

## Data Availability

The datasets generated and analysed during the current study are available from the corresponding author on reasonable request.
