# Supplementary material for: Short course of antimicrobial treatment for uncomplicated enterococcal bacteremia
Source: Eur J Clin Microbiol Infect Dis. 2025 Nov 7;45(2):557–65. doi: 10.1007/s10096-025-05348-6 (PMC12987813; doi:10.1007/s10096-025-05348-6)
Supplement: Supplementary file 1 — Supplementary Material 1 [file 10096_2025_5348_MOESM1_ESM.docx]

**Supplementary Table 1.** Antibiotic treatment details

|  | **Short duration**  **(n=138)** | **Long duration (n=193)** |
| --- | --- | --- |
| Targeted intravenous therapy | n=138 | n=193 |
| Amoxicillin | 21 (15) | 62 (32) |
| Co-amoxicillin | 22 (16) | 33 (17) |
| Piperacillin/tazobactam | 24 (17) | 23 (12) |
| Carbapenem | 13 (9) | 5 (3) |
| Vancomycin | 58 (42) | 68 (35) |
| Other | 2 (1) | 7 (4) |
| Step-down oral treatment | n=15 | n=51 |
| Amoxicillin | 3 (20) | 24 (47) |
| Co-amoxicillin | 11 (73) | 23 (45) |
| Other | 1 (7) | 4 (8) |

Data are depicted as number (%)

**Supplementary Table 2.** Diagnoses of episodes with recurrent bacteremia caused by the same enterococcal species within 120 days of the initial episode

|  | **Species** | **Diagnosis of the initial episode** | **Diagnosis of the subsequent episode** |
| --- | --- | --- | --- |
| 1 | *E. faecalis* | Urinary-tract | Spondylodiscitis |
| 2 | *E. faecalis* | Urinary-tract | Spondylodiscitis |
| 3 | *E. faecalis* | Cholangitis | Cholangitis |
| 4 | *E. faecalis* | Cholangitis | Cholangitis |
| 5 | *E. faecalis* | Cholangitis | Cholangitis |
| 6 | *E. faecalis* | Cholangitis | Cholangitis |
| 7 | *E. faecalis* | Cholangitis | Cholangitis |
| 8 | *E. faecalis* | Cholangitis | Cholangitis |
| 9 | *E. faecium* | Cholangitis | Cholangitis |
| 10 | *E. faecium* | Cholangitis | Cholangitis |
| 11 | *E. faecium* | Cholangitis | Cholangitis |
| 12 | *E. faecium* | Cholangitis | Cholangitis |

**Supplementary Table 3.** Comparison of primary outcomes of episodes with short (4-10 days) and long duration of antimicrobial treatment (11-18 days) among 187 episodes with bacteremia by *E. faecalis* and 155 episodes with bacteremia by other enterococcal species

|  | **Bacteremia by *E. faecalis*** | | | **Bacteremia by other enterococcal species** | | |
| --- | --- | --- | --- | --- | --- | --- |
|  | **Short duration**  **(n=64)** | **Long duration**  **(n=123)** | ***P*** | **Short duration**  **(n=78)** | **Long duration**  **(n=77)** | ***P*** |
| Primary endpoint at 120 days | 15 (23) | 26 (21) | 0.714 | 19 (24) | 19 (25) | 1.000 |
| Mortality | 12 (19) | 21 (17) | 0.840 | 19 (24) | 19 (25) | 1.000 |
| Recurrence of bacteremia with the same organism | 4 (5) | 5 (4) | 1.000 | 1 (1) | 3 (4) | 0.367 |
| New bone and joint infection | 1 (2) | 1 (1) | 1.000 | 0 (0) | 0 (0) | - |

Data are depicted as number (%)
